# Eco-Virological Preliminary Study of Potentially Emerging Pathogens in Hedgehogs (*Erinaceus europaeus*) Recovered at a Wildlife Treatment and Rehabilitation Center in Northern Italy

**DOI:** 10.3390/ani10030407

**Published:** 2020-03-01

**Authors:** Mauro Delogu, Claudia Cotti, Davide Lelli, Enrica Sozzi, Tiziana Trogu, Antonio Lavazza, Giacomo Garuti, Maria Rita Castrucci, Gabriele Vaccari, Maria Alessandra De Marco, Ana Moreno

**Affiliations:** 1 Department of Veterinary Medical Sciences, University of Bologna, 50 Via Tolara di Sopra, 40064 Ozzano dell’Emilia (BO), Italy; mauro.delogu@unibo.it (M.D.); claudia.cotti@unibo.it (C.C.); giacomo.garuti@studio.unibo.it (G.G.); 2 Virology Unit, Istituto Zooprofilattico Sperimentale della Lombardia e dell’Emilia Romagna, 7/9 Via Bianchi, 25124 Brescia, Italy; davide.lelli@izsler.it (D.L.); enrica.sozzi@izsler.it (E.S.); tiziana.trogu@izsler.it (T.T.); antonio.lavazza@izsler.it (A.L.); anamaria.morenomartin@izsler.it (A.M.); 3 Department of Infectious Diseases, Istituto Superiore di Sanità, 299 Viale Regina Elena, 00161 Rome, Italy; mariarita.castrucci@iss.it; 4 Department of Food Safety, Nutrition and Veterinary Public Health, Istituto Superiore di Sanità, 299 Viale Regina Elena, 00161 Rome, Italy; gabriele.vaccari@iss.it; 5 Wildlife Service, Institute for Environmental Protection and Research (ISPRA), 9 Via Ca’ Fornacetta, 40064 Ozzano dell’Emilia (BO), Italy

**Keywords:** wild animals, hedgehogs, public health, coronaviruses, *Erinaceus* coronavirus (EriCoV), Betacoronavirus infection

## Abstract

**Simple Summary:**

Most of the newly emerging infections arise from animal reservoirs, frequently represented by wildlife species. Western European hedgehogs (*Erinaceus europaeus*) are mammalian hibernators, mainly nocturnal and insectivorous, living in natural open and green spaces as well as artificial, rural and urban, areas. They are generalist predators of macro-invertebrates, but they may also eat meat, bird eggs and on occasion pet food. These ecological and feeding habits, along with their high population densities, notable synanthropic attitudes, frequent contacts with sympatric wild and domestic species, including humans, implicate the possibility of intra- and interspecies interactions accounting for the possible involvement of *E. europaeus* in the ecology of several potentially emerging pathogens, including coronaviruses. Using PCR-based and virus isolation methods, we found that 58.3% of 24 hedgehogs’ fecal samples were PCR-positive for *Erinaceus* coronaviruses (EriCoVs). We did not observe any clinical disease related to the EriCoV infection in hedgehogs. However, the high mutation rates characterizing members of the *Coronaviridae* family and their potential successful interspecies host jumps—as that likely occurred in the Novel coronavirus (2019-nCoV) emergence—should be considered in the management of hedgehogs admitted to multi-species wildlife rehabilitation centers, recommending their return back to the original recovery areas.

**Abstract:**

The Western European Hedgehog (*Erinaceus europaeus*) is one of the four hedgehog species belonging to the genus *Erinaceus*. Among them, *E. amurensis* is extant in East Asia’s areas only, whereas *E. europaeu*s, *E. roumanicus* and *E. concolor* are mainly found in Europe. *E. europaeus* is endemically distributed from western to central and southern Europe, including Italy. Western European hedgehogs’ ecological and feeding habits, along with their high population densities, notable synanthropic attitudes, frequent contacts with sympatric wild and domestic species, including humans, implicate the possible involvement of *E. europaeus* in the ecology of potentially emerging viruses, such as coronaviruses, influenza A and influenza D viruses, canine distemper virus, pestiviruses and Aujeszky’s disease virus. We examined 24 *E. europaeus* individuals found injured in urban and rural areas of Northern Italy. Of the 24 fecal samples collected and tested for the above-mentioned pathogens by both PCR-based and virus isolation methods, 14 were found PCR-positive for betacoronaviruses belonging to lineage C and related to the known *Erinaceus* coronaviruses (EriCoVs), as determined by partial sequencing of the virus genome. Our findings suggest that hedgehogs could be considered natural reservoirs of CoVs, and also act as chronic shedding carriers of these potentially emerging RNA viruses.

## 1. Introduction

Western European Hedgehog (*Erinaceus europaeus*) is one of the four hedgehog species belonging to the genus *Erinaceus*, biologically classified in the order *Eulipotyphla* [1]. Among them, *E. amurensis* is extant in East Asia’s areas only, whereas *E. europaeus*, *E. roumanicus* and *E. concolor* are mainly found in Europe. In particular, *E. europaeus*—from here on also simply called hedgehog—is a terrestrial mammal endemically distributed from western to central and southern Europe; this species is also present in parts of Fennoscandia, in north-western European Russia, the British Isles, Azores and several Mediterranean islands [2]. *E. europaeus*, protected by the Italian law 157/92 [3], is abundantly distributed throughout the Italian peninsula, in Sicily, in Sardinia and in some smaller islands, without any reported evidence of a rapid decline [4].

Hedgehogs are mammalian hibernators, with different plesiomorphic features in their morphology, physiology and behavior. They are mainly nocturnal and insectivorous, build nests and use hearing and smell as a dominant sense, and possess peculiar specializations such as spines and highly developed back muscles allowing them to roll up [5]. Hedgehogs’ suitable habitats include natural open and green spaces as well as artificial, rural and urban, areas with marked preference for lowlands and hilly areas providing abundant food supply and plenty of grass, trees and fallen leaves. They also need diversity of habitats as those found in edges of deciduous woodlands or in man-made ecotonal interfaces (i.e., parks and garden with hedges) [6]. Despite its solitary lifestyle [7], *E. europaeus* has a highly variable home range, ranging in Italy from 5.5 to 102.5 ha [8]. As generalist predators of macro-invertebrates, hedgehogs consume most frequently caterpillars, earthworms, earwigs, slugs, millipedes and beetles (including coprophagous species); they may also eat meat (e.g., in the form of carrion or preyed small vertebrates such as mice, snakes and birds), bird eggs and pet food that is frequently found in gardens and shared with dogs and cats.

The above-mentioned ecological and feeding habits, along with their high population densities, notable synanthropic attitudes and frequent contacts with sympatric wild and domestic species, including humans [5], implicate intra- and interspecies interactions [9] accounting for the potential involvement of *E. europaeus* (e.g., as a maintenance host, bridge host or dead-end host) in the ecology of several potentially emerging pathogens, as those reported below.

Coronaviruses (CoVs) can infect productively a wide range of animal species and cause respiratory, enteric and neurological diseases of variable severity. As shown during the last two decades, potentially zoonotic CoVs can pose massive public health threats such as those related to the Severe Acute Respiratory Syndrome Coronavirus (SARS-CoV) [10], Middle East Respiratory Syndrome Coronavirus (MERS-CoV) [11] and 2019 Novel Coronavirus (2019-nCoV) [12] emergences. Two of the four recognized genera of CoVs (i.e., *Alphacoronavirus* and *Betacoronavirus*) are probably perpetuated in bat populations [13,14], recently recognized as the evolutionary origins of the MERS-CoV, emerged in 2012 in humans [15]. The recent detection of MERS-related CoVs in hedgehogs from Germany [16], France [17] and Great Britain [18] suggests that *E. europaeus*, which is belonging to a *Chiroptera*-related order [19], represents a wild reservoir of betacoronaviruses known as *Erinaceus* CoVs (EriCoVs).

The wild bird *Influenza A virus* (IAV) gene pool poses significant risks for both animal and human health because of its ability to colonize a wide variety of animal species (included in the *Mammalia*, *Aves* and *Reptilia* classes) in which IAV can cause variable outcomes of infection, with possible high morbidity and fatality rates [20]. The newly identified *Influenza D virus* (IDV), also circulating in Italian livestock [21], is known to infect cattle, which are the main reservoir of the virus, and small ruminants, swine, horses, camels, ferrets and humans. Both IAV and IDV exhibit respiratory and fecal shedding and the broad IAV host range includes wild and pet species sympatric or potentially sympatric with *E. europaeus* (e.g., dogs, cats, ferrets, land-based birds) [22].

*Canine morbillivirus* (canine distemper virus, CDV) causes a highly contagious and systemic disease often involving the respiratory, gastro-intestinal and nervous systems of canids and poses worldwide a relevant health risk for domestic and wild species of carnivores [23,24,25]. Its wide range of naturally infected hosts includes *Rodentia*, *Primates*, *Artiodactyla*, *Proboscidea* and *Eulipotyphla* species, and a morbillivirus antigenically related to CDV was also isolated in fecal samples obtained from a sick *E. europaeus* [26].

Pestiviruses include eleven known *Pestivirus* species along with related unclassified emerging viruses that can infect domestic and wild ruminants, pigs and wild boars [27], rats and bats. Pestiviruses can cause a wide variety of symptoms ranging from mild to severe disease and even death. Transmission of pestivirus infection can occur through nasal secretion, urine, feces, contact with fomites and by vertical routes [28]. To the best of our knowledge, no pestivirus infection has been ever reported in hedgehogs.

*Suid alphaherpesvirus 1* (SHV-1) is the etiological agent of the Aujeszky’s disease and may infect a very broad range of domestic and wild mammalian species, including hedgehogs [29], but not humans and tailless apes. SHV-1 replicates in the central nervous system and other organs, such as the respiratory tract, and only pigs and wild boars can survive productive infections, thus playing the role of a natural reservoir in which the virus can persist latently after clinical recovery [30]. Occasional consumption of wild boar carrions could expose hedgehogs to SHV-1 infection, which is quite frequently detected in populations of this wild suid species in Italy [31].

The aim of this study is to investigate the presence of the above-mentioned viral pathogens in fecal samples of hedgehogs to better clarify the role of *E. europaeus* in the ecology of such viruses. This research could also provide epidemiological information useful for the health management of hedgehogs admitted to wildlife rescue centers, where different animal species, including humans, can interact.

## 2. Materials and Methods

### 2.1. Sample Collection

Fecal material was collected non-invasively (i.e., by collecting freshly deposited feces, avoiding manipulation of the animals) from 24 hedgehogs found injured in both urban areas (cities, towns, villages and their suburbs) and rural areas in three provinces of the Emilia-Romagna region (Northern Italy) (Table 1), and then recovered at a wildlife treatment and rehabilitation center (WTRC). When admitted to the WTRC, hedgehogs were kept in isolation until sample collection (within 4 days of admission) to reduce the possibility of nosocomial infections. Between November 2018 and January 2019, the samples were collected individually and stored in transport medium (1:1 PBS:glycerol with potassium penicillin, streptomycin sulfate, gentamicin sulfate, polymyxin B, mycostatin) at −20 °C until laboratory examinations.

### 2.2. Virological Investigation

#### 2.2.1. Molecular and Virus Isolation Assays

Different real time PCRs were performed to detect IAV [32], IDV [33], CDV [34], and SHV-1 [35]. Pestiviruses were tested by a pan-pestivirus assay targeting most of known species and related unclassified viruses [36]. A pan-coronavirus nested PCR for the RNA-dependent RNA polymerase (RdRp) gene was carried out to obtain a 440 bp PCR amplicon [37], which was sequenced by using a Big-Dye Terminator Cycle sequencing kit (Applied Biosystems, Foster City, CA, USA) and the same primers used for amplification. Sequencing was performed using the inner primers only and the final PCR product analyzed by sequencing was 440 bp long. Positive and negative controls used were the BetaCoV bovine field strain Bov/Italy/21562/2019 and feces from SPF chickens, respectively.

Virus isolation was attempted by inoculation of fecal samples in confluent monolayers of VERO cells BS CL 86 (African green monkey kidney) according to Lelli et al. [38].

#### 2.2.2. Statistical Analysis

Fisher’s exact test was used to test significant differences in virus-detection prevalence between urban and rural areas (EPISTAT 3.3, Epistat Services, Richardson, TX, USA).

#### 2.2.3. Phylogenetic Analysis

Phylogenetic tree was constructed using the IQ-TREE 1.6.9 software under the TIM2 + F + G4 model with a bootstrap analysis of 1000 replicates [39]. Only bootstrap values >50% were reported. The CoV RdRp gene sequences obtained from Italian hedgehogs (*E. europaeus* species) were compared with those originated from hedgehogs (including *E. europaeus* and *E. amurensis* sequences), bats, humans and camels. Sequences were named according to a pattern including: GenBank accession number, virus name, virus strain, host species, country of isolation and year of isolation.

## 3. Results

### 3.1. Virological Investigation

#### 3.1.1. Molecular and Virus Isolation Assays

Of the 24 hedgehogs examined, 14 (58.3%) tested positive for *Erinaceus* CoVs, named EriCoVs, by RT-PCR: attempts of isolation on cell culture did not give any positive results. All samples were negative for influenza A and influenza D viruses, canine distemper virus, pestiviruses and Aujeszky’s disease virus when tested by both molecular and virus isolation methods.

#### 3.1.2. Statistical Analysis

No significant difference was found between percentages of hedgehogs found to be CoV-positive in rural (80%) and urban (52.6%) areas.

#### 3.1.3. Phylogenetic Analysis

**Figure 1:**
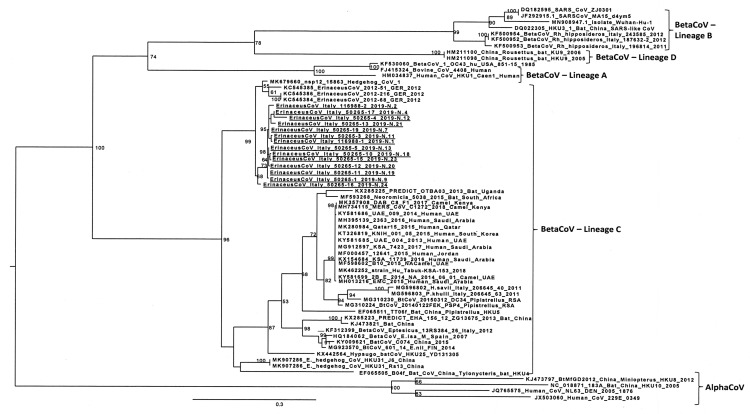
Maximum likelihood phylogenetic tree of lineage C betacoronaviruses, based on partial nucleotide sequences of the CoV RNA-dependent RNA polymerase gene. The EriCoVs detected in the present study are underlined (see Table 1 for details).

Phylogentic analysis was based on partial sequencing of RdRp gene of Italian sequences compared with those of alpha and betaCoVs originated from hedgehogs, bats, humans and camels. Results showed that EriCoVs from Italian hedgehogs (Figure 1) grouped together with MERS-like betacoronaviruses obtained from Western European hedgehogs sampled in Germany and Great Britain (>92.1% of nucleotide identity) [16,18], forming a subclade within the BetaCoV lineage 2C. This lineage, recently renamed as *Merbecovirus* subgenus [14], included MERS and MERS-like CoV sequences. In particular, the Italian hedgehog viruses were related to MERS-CoV, showing an identity from 79.9% to 81.2% with some MERS-CoV representative strains.

## 4. Discussion

Since the first detection of a novel *Betacoronavirus* in hedgehogs [16], *E. europaeus* has been indicated as a possible additional wild reservoir of emerging MERS-like CoVs with potential public health implications. In the present study, an overall prevalence of EriCoV infection (58.3%) was detected in sampled hedgehogs with no significant difference between percentages found in rural (80%) and urban (52.6%) areas. Indeed, this may be associated with a sustained circulation of viruses in their wild recovery areas likely due to frequent and consistent overlaps of home ranges reported in this species [8]. The detected prevalence was higher than that observed by Saldanha et al. [18] in Great Britain (10.8%), but in accordance with data reported in Germany (58.9%) and France (50%), respectively [16,17]. From an epidemiological point of view, the high EriCov prevalence observed suggests that, in the study area, *E. europaeus* could play the role of natural reservoir, competent for infection, replication and excretion of this virus. Such high prevalence observed in this study also suggests that hedgehogs could represent chronic shedding carriers of the virus, as reported for alphacoronaviruses infecting bats [40] and cats [41]. It is also noteworthy that most of our samples were collected during a physiological phase of reduced immune function [42] that occurs during hedgehogs’ hibernation period, consisting of four or five months of “winter dormancy” in which these mammals may alternate phases of sleep and arousal [5].

As previously reported [16,18], we did not observe any clinical disease related to this infection in hedgehogs, that were all reintroduced into the wild within April 2019. Nevertheless, the high mutation rates characterizing members of the *Coronaviridae* family and their possible successful interspecies host jumps [43] should be considered in the management of hedgehogs admitted to multi-species wildlife rehabilitation centers [44], recommending their return back to the original recovery areas.

Phylogenetic analysis showed that EriCoVs from *E. europaeus* clustered together, whereas, interestingly, novel betacoronavirus sequences, recently detected in Amur Hedgehogs from China [45], were placed in a different group (nucleotide identity to *E. europaeus* sequences ranging from 82.9% to 85.6%) within the same lineage, closely related to bat BetaCoV HKU4 detected in China.

A limit of this study is that we cannot definitively exclude hedgehogs’ involvement in the ecology of the viral pathogens that tested negative. In fact, the prevalence detection threshold value, which is related to the sample size (24 hedgehogs), did not allow us to be 95% certain of detecting at least one positive sample, when the virus circulation prevalence was lower than 15% [46].

Future studies will be pointed to increase the number of sampled hedgehogs as well as expand the number of urban and rural areas sampled in order to strengthen the results here reported.

## 5. Conclusions

Overall, we provide new information about distribution areas of EriCoV infection in *E. europaeus* populations. Our preliminary observations suggest that this species might act as a reservoir for EriCoV in Northern Italy’s areas. However, future research will be needed before confirming the role of *E. europaeus* as competent reservoir of EriCoV. A more in-depth molecular analysis, based on a whole genome characterization, will also be required to provide more information about EriCoVs from Italy.

## Figures and Tables

**Table 1:** Detection of *Erinaceus* coronaviruses (EriCoVs) in Western European hedgehogs (*Erinaceus europaeus*) from rural and urban areas of Northern Italy.

Sample ID	Date of Submission to WTRC	Municipality or Hamlet of Recovery (Province)	Recovery Site	Pan-CoV RT-PCR	^a^ GenBank Accession Number
**ER-1/116988-1**	11/10/18	Budrio (BO)	R.A.	+	MT024739
**ER-2/116988-2**	11/10/18	Bologna (BO)	U.A.	+	MT024740
ER-3	11/11/18	Bologna (BO)	U.A.	-	nd
**ER-4/50265-17**	11/19/18	Bologna (BO)	U.A.	+	MT024741
ER-5	11/19/18	Casalecchio (BO)	U.A.	-	nd
ER-6	11/09/18	Bologna (BO)	U.A.	-	nd
**ER-7/50265-19**	11/29/18	Bologna (BO)	U.A.	+	MT024742
ER-8	11/30/18	Bologna (BO)	U.A.	-	nd
**ER-9/50265-1**	12/14/18	Bentivoglio (BO)	R.A.	+	MT024743
ER-10	12/14/18	Bologna (BO)	U.A.	-	nd
**ER-11/50265-3**	12/16/18	Sala Bolognese (BO)	U.A.	+	MT024744
**ER-12/50265-4**	12/18/18	Bologna (BO)	U.A.	+	MT024745
**ER-13/50265-5**	12/20/18	Minerbio (BO)	U.A.	+	MT024746
ER-14	12/20/18	Lugo (RA)	U.A.	-	nd
ER-15	12/29/18	Copparo (FE)	U.A.	-	nd
ER-16	12/29/18	Bologna (BO)	U.A.	-	nd
ER-17	12/30/18	Cona (FE)	R.A.	-	nd
**ER-18/50265-10**	12/30/18	Cona (FE)	R.A.	+	MT024747
**ER-19/50265-11**	01/04/19	Cona (FE)	R.A.	+	MT024748
**ER-20/50265-12**	01/04/19	Bentivoglio (BO)	U.A.	+	MT024749
**ER-21/50265-13**	01/05/19	Granarolo (BO)	U.A.	+	MT024750
ER-22	01/09/19	Bologna (BO)	U.A.	-	nd
**ER-23/50265-15**	01/11/19	Imola (BO)	U.A.	+	MT024751
**ER-24/50265-16**	13/01/19	Bentivoglio (BO)	U.A.	+	MT024752

WTRC, wildlife treatment and rehabilitation center; ER; Emilia-Romagna; BO, Bologna; RA, Ravenna,; FE, Ferrara; R.A., rural area; U.A., urban area; Pan-CoV, Pan-coronavirus; RT-PCR, reverse transcriptase-PCR; nd, not done; +, positive; -, negative. Bold ID indicates a positive sample. ^a^ GenBank accession number assigned to partial RNA-dependent RNA polymerase gene sequences of CoV strains found in hedgehogs.
